# Safety Monitoring of COVID-19 Vaccine Booster Doses Among Adults — United States, September 22, 2021–February 6, 2022

**DOI:** 10.15585/mmwr.mm7107e1

**Published:** 2022-02-18

**Authors:** Anne M. Hause, James Baggs, Paige Marquez, Tanya R. Myers, John R. Su, Phillip G. Blanc, Jane A. Gwira Baumblatt, Emily Jane Woo, Julianne Gee, Tom T. Shimabukuro, David K. Shay

**Affiliations:** ^1^CDC COVID-19 Emergency Response Team; ^2^Food and Drug Administration, Silver Spring, Maryland.

During September 22, 2021–February 6, 2022, approximately 82.6 million U.S. residents aged ≥18 years received a COVID-19 vaccine booster dose.* The Food and Drug Administration (FDA) has authorized a booster dose of either the same product administered for the primary series (homologous) or a booster dose that differs from the product administered for the primary series (heterologous). These booster authorizations apply to all three COVID-19 vaccines used in the United States (*1*–*3*).^†^ The Advisory Committee on Immunization Practices (ACIP) recommended preferential use of an mRNA COVID-19 vaccine (mRNA-1273 [Moderna] or BNT162b2 [Pfizer-BioNTech]) for a booster, even for persons who received the Ad26.COV2.S (Janssen [Johnson & Johnson]) COVID-19 vaccine for their single-dose primary series.^§^ To characterize the safety of COVID-19 vaccine boosters among persons aged ≥18 years during September 22, 2021–February 6, 2022, CDC reviewed adverse events and health impact assessments following receipt of a booster that were reported to v-safe, a voluntary smartphone-based safety surveillance system for adverse events after COVID-19 vaccination, and adverse events reported to the Vaccine Adverse Event Reporting System (VAERS), a passive vaccine safety surveillance system managed by CDC and FDA. Among 721,562 v-safe registrants aged ≥18 years who reported receiving a booster, 88.8% received homologous COVID-19 mRNA vaccination. Among registrants who reported a homologous COVID-19 mRNA booster dose, systemic reactions were less frequent following the booster (58.4% [Pfizer-BioNTech] and 64.4% [Moderna], respectively) than were those following dose 2 (66.7% and 78.4%, respectively). The adjusted odds of reporting a systemic reaction were higher following a Moderna COVID-19 vaccine booster, irrespective of the vaccine received for the primary series. VAERS has received 39,286 reports of adverse events after a COVID-19 mRNA booster vaccination for adults aged ≥18 years, including 36,282 (92.4%) nonserious and 3,004 (7.6%) serious events. Vaccination providers should educate patients that local and systemic reactions are expected following a homologous COVID-19 mRNA vaccine booster; however, these reactions appear less common than those following dose 2 of an mRNA-based vaccine. CDC and FDA will continue to monitor vaccine safety and provide data to guide vaccine recommendations and protect public health.

V-safe (https://vsafe.cdc.gov/en/) is a voluntary, smartphone-based U.S. safety surveillance system established to monitor adverse events after COVID-19 vaccination. The platform allows existing registrants to report receiving a COVID-19 booster dose and new registrants to enter information about all COVID-19 vaccine doses received. Health surveys are sent daily during the first week after receipt of each dose and include questions about local injection site and systemic reactions and health impacts.^¶^ CDC’s v-safe call center contacts registrants who indicate that medical care was sought after vaccination and encourages completion of a VAERS report, if indicated.

VAERS is a U.S. national passive vaccine safety surveillance system managed by CDC and FDA that monitors adverse events after vaccination (*4*). VAERS accepts reports from health care providers, vaccine manufacturers, and members of the public.** VAERS reports are classified as serious if there are any reports of hospitalization, prolongation of hospitalization, life-threatening illness, permanent disability, congenital anomaly or birth defect, or death.^††^ VAERS staff members assign Medical Dictionary for Regulatory Activities (MedDRA) preferred terms to the signs, symptoms, and diagnostic findings in VAERS reports.^§§^ Previous reports of myocarditis and pericarditis following receipt of COVID-19 vaccine were identified by a search for selected MedDRA preferred terms (*5*); CDC staff members attempted to collect information from health care providers about clinical course and determined whether the case definition for myocarditis or pericarditis was met.^¶¶^

Local and systemic reactions and health impacts reported during the week following booster vaccination were described for v-safe registrants aged ≥18 years who received a COVID-19 booster (≥2 months after a single dose of Janssen COVID-19 vaccine or ≥5 months after the second dose of a COVID-19 mRNA vaccine) during September 22, 2021–February 6, 2022, and completed at least one v-safe health check-in survey in the week after each vaccination. Registrants who reported receiving a COVID-19 mRNA primary vaccination series followed by a Janssen booster (476) were excluded from the analysis because of small numbers. VAERS reports for persons aged ≥18 years who received a COVID-19 mRNA vaccine booster during September 22, 2021–February 6, 2022, were described by severity (serious versus nonserious), demographic characteristics (i.e., age, sex, race, and ethnicity), and MedDRA preferred terms. Reporting rates for myocarditis reports meeting the case definition after a booster were stratified by sex and age group. Multivariable analyses were conducted to estimate the adjusted odds of reporting an adverse event or health impact by comparing 1) dose 2 and booster for registrants who received homologous COVID-19 mRNA vaccination, and 2) homologous and heterologous booster vaccination. SAS software (version 9.4; SAS Institute) was used to conduct all analyses.*** These surveillance activities were reviewed by CDC and conducted consistent with applicable federal law and CDC policy.^†††^

## Review of v-safe Data

During September 22, 2021–February 6, 2022, a total of 721,562 unique v-safe registrants aged ≥18 years reported having received a COVID-19 vaccine booster; 640,586 (88.8%) reported homologous COVID-19 mRNA vaccination. Among 307,998 registrants who reported a homologous Moderna booster, local and systemic reactions were less frequently reported during the week following booster (71.8% and 64.4%, respectively) than following dose 2 (81.4% and 78.4%, respectively) (p<0.001) (Figure). Among 332,588 registrants who reported a homologous Pfizer-BioNTech booster, local and systemic reactions were also reported less frequently following the booster (64.3% and 58.4%, respectively) than following dose 2 (68.1% and 66.7%, respectively) (p<0.001). Health impacts, including inability to perform daily activities and inability to work, were also reported less frequently following dose 2. Among homologous Moderna booster recipients, receipt of medical care was reported more frequently following the booster (0.8%) than dose 2 (0.7%); however, the difference was not significant (p = 0.06). Among homologous Pfizer-BioNTech booster recipients, receipt of medical care was reported significantly (p<0.001) more frequently following the booster (0.9%) than following dose 2 (0.6%). All registrants who indicated that medical care was sought after vaccination were contacted and encouraged to complete a VAERS report.

**FIGURE:**
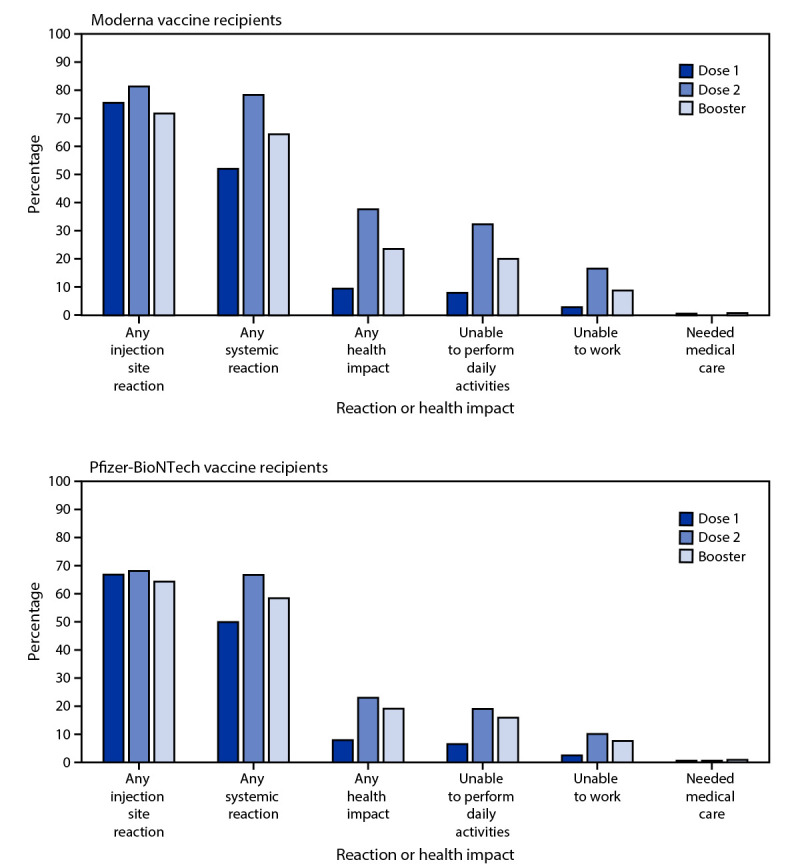
Adverse reactions and health impacts* reported by adults aged ≥18 years who received a homologous Moderna (N = 307,998) or Pfizer-BioNTech (N = 332,588) COVID-19 vaccine booster and completed at least one v-safe health check-in survey on days 0–7 after each vaccine dose, by dose — United States, September 22, 2021–February 6, 2022 * Local injection site reactions include itching, pain, redness, and swelling. Systemic reactions include abdominal pain, myalgia, chills, diarrhea, fatigue, fever, headache, joint pain, nausea, rash, and vomiting. Health impacts include inability to perform normal daily activities, inability to work or attend school, and receipt of medical care. The odds of reporting any local injection site or systemic reaction or health impact following dose 2 and booster dose were compared using a multivariable generalized estimating equations model that accounted for the correlation between registrants and adjusted for demographic variables; p<0.01 was considered statistically significant. All dose 2 and booster dose comparisons were statistically significant, except receipt of medical care among homologous Moderna COVID-19 vaccine recipients.

Among primary Moderna, Pfizer-BioNTech, and Janssen series v-safe registrants, 94.1%, 95.1%, and 17.2%, respectively, received a homologous booster. Among primary mRNA series vaccine v-safe registrants, 5.9% of Moderna and 4.8% of Pfizer-BioNTech registrants received a heterologous mRNA vaccine booster. Among primary Janssen v-safe registrants, 52.3% received a Moderna booster, and 30.5% received a Pfizer-BioNTech booster (Table 1). Among registrants who received a Moderna COVID-19 vaccine primary series, the odds of reporting a systemic reaction (calculated using a logistic regression model that adjusted for demographic variables) were lower among those who reported a heterologous Pfizer-BioNTech vaccine booster than among those who reported a homologous Moderna COVID-19 vaccine booster (adjusted odds ratio [aOR] = 0.85; 95% CI = 0.82–0.88). Among v-safe registrants who received a Pfizer-BioNTech or Janssen primary series, the odds of reporting a systemic reaction were higher among those who received a heterologous Moderna vaccine booster than among those who received a homologous COVID-19 vaccine booster (aOR = 2.24; 95% CI = 2.14–2.33 [Pfizer-BioNTech primary series recipients] and OR = 1.08; 95% CI = 1.03–1.14 [Janssen primary series recipients]). 

**TABLE 1:** Adjusted odds ratios* and 95% CI for reactions and health impacts following homologous or heterologous COVID-19 vaccine booster dose among adults aged ≥18 years, by primary vaccination series and booster vaccine product received (N = 721,562) — United States, September 22, 2021–February 6, 2022

Primary series/Booster vaccine (no.)	No. of booster doses (%)	Reaction^†^ (%)		
		Any injection site reaction	Any systemic reaction	Any health impact
**Moderna^§^ (n = 327,464)**				
Moderna	307,998 (94.1)	71.8	64.4	23.6
Pfizer-BioNTech	19,222 (5.9)	70.7	66.7	23.4
aOR (95% CI)	—	0.70 (0.68–0.73)^¶^	0.85 (0.82–0.88)^¶^	0.81 (0.78–0.84)^¶^
**Pfizer-BioNTech^§^ (n = 349,545)**				
Pfizer-BioNTech	332,588 (95.1)	64.3	58.4	19.1
Moderna	16,725 (4.8)	87.7	82.9	39.5
aOR (95% CI)	—	2.41 (2.30–2.53)^¶^	2.24 (2.14–2.33)^¶^	2.06 (1.99–2.13)^¶^
Janssen** **(n = 44,553)**				
Janssen	7,656 (17.2)	52.3	56.2	16.6
Moderna	23,310 (52.3)	65.9	58.2	19.0
OR (95% CI)	—	1.76 (1.67–1.86)^¶^	1.08 (1.03–1.14)^¶^	1.18 (1.10–1.26)^¶^
Pfizer-BioNTech	13,587 (30.5)	62.0	56.6	16.8
OR (95% CI)	—	1.49 (1.41–1.57)^¶^	1.01 (0.96–1.07)	1.01 (0.94–1.09)

**Abbreviations:** aOR = adjusted odds ratio; OR = odds ratio.

* Includes persons who completed at least one v-safe health check-in survey on days 0–7 after receipt of each vaccine dose. The odds of reporting an event following homologous (referent group) and heterologous booster vaccination were compared using a logistic regression model that adjusted for demographic variables (i.e., age, sex, race, and ethnicity) of registrants. Odds ratios were not adjusted for persons who reported a primary Janssen series because of small numbers.

^†^ Local injection site reactions include itching, pain, redness, and swelling. Systemic reactions include abdominal pain, myalgia, chills, diarrhea, fatigue, fever, headache, joint pain, nausea, rash, and vomiting. Health impacts include inability to perform normal daily activities, inability to work or attend school, and receipt of medical care.

^§^ The model did not converge for the 476 registrants who reported COVID-19 mRNA vaccination primary dose followed by a Janssen booster, and they were excluded from the analysis.

^¶^ P<0.01 was considered statistically significant.

** Includes persons who received a primary Janssen single-dose and one additional dose of vaccine from the listed manufacturers.

## Review of VAERS Data

During September 22, 2021–February 6, 2022, VAERS received and processed 39,286 reports of adverse events following receipt of a COVID-19 mRNA vaccine booster for persons aged ≥18 years; the median age was 54 years, and 25,966 (66.1%) reports were in women. Most VAERS reports were for nonserious events (36,282; 92.4%); the most commonly reported conditions were headache (5,237; 13.3%), fever (5,194; 13.2%), and pain (4,931; 12.6%). Among 37 reports of myocarditis that met the case definition, 26 (70.3%) were in men, and the median patient age was 32 years. The reporting rate for myocarditis (Table 2) was highest among men aged 18–24 years following Moderna COVID-19 vaccine booster (8.7 per 1 million doses administered). One person with myocarditis reported to VAERS after COVID-19 vaccination died; investigation of this death is ongoing and to date has not eliminated other potential contributory factors.

**TABLE 2:** Cases and rates* of myocarditis reported to the Vaccine Adverse Event Reporting System^†^ following receipt of an mRNA COVID-19 booster dose among adults aged ≥18 years (N = 37), by age, sex, and vaccine product received — United States, September 22, 2021–February 6, 2022

Age group, yrs	No. of cases (rates)*^,§^			
	Pfizer-BioNTech (n = 18)		Moderna (n = 18)	
	Men (n = 16)	Women (n<5)	Men (n = 10)	Women (n = 8)
18–24	5 (4.1)	<5 (<1.0)	6 (8.7)	<5 (1.1)
25–29	<5 (1.1)	0 (—)	<5 (3.2)	<5 (1.2)
30–39	<5 (1.7)	<5 (<1.0)	<5 (<1.0)	<5 (1.5)
40–49	0 (—)	0 (—)	0 (—)	<5 (<1.0)
50–64	<5 (<1.0)	0 (—)	0 (—)	<5 (<1.0)
≥65^¶^	5 (<1.0)	0 (—)	<5 (<1.0)	0 (—)

**Abbreviations:** MedDRA = Medical Dictionary for Regulatory Activities; VAERS = Vaccine Adverse Event Reporting System.

* Cases per 1 million doses administered.

**^†^** VAERS reports of myocarditis were identified using a combination of MedDRA preferred terms, with symptom onset during day of vaccination through day 6 after vaccination and verified to meet case definition by clinician interview with a health care provider, or clinician review of the medical record. The analysis includes persons receiving both homologous and heterologous booster doses.

^§^ Cells with fewer than two persons were suppressed and indicated as “<5” for confidentiality.

^¶ ^Includes one report with sex of patient not reported.

## Discussion

Among 721,562 v-safe registrants aged ≥18 years who reported receiving a COVID-19 vaccine booster, most received homologous COVID-19 mRNA vaccination. Similar to findings from Moderna and Pfizer-BioNTech clinical trials (*6*,*7*), observational v-safe data demonstrated that local and systemic reactions were reported less frequently following a homologous booster dose than after receipt of the second COVID-19 mRNA vaccine dose. Medical care was rarely sought; however, registrants reported care significantly more frequently following administration of Pfizer-BioNTech COVID-19 booster vaccination than after Pfizer-BioNTech COVID-19 dose 2. V-safe does not capture diagnoses associated with hospitalization; however, registrants can include supplemental text for each health check-in. Whether hospitalization was the result of vaccination could not be determined from v-safe data; however, all registrants who reported hospitalization were contacted and encouraged to complete a VAERS report.

A recently published study evaluating the immunogenicity and safety of heterologous booster vaccinations for all COVID-19 vaccines authorized in the United States found that reports of adverse events were similar, regardless of the type of booster received; however, the sample size was not large enough to compare small differences in risk.^§§§^ In v-safe, heterologous boosters were infrequently reported; however, the odds of reporting a systemic reaction were higher following a Moderna COVID-19 vaccine booster, irrespective of the primary series received. This finding is consistent with reactions reported to v-safe following Moderna primary series vaccination (*8*). The adjusted odds ratios appear to differ qualitatively from the raw frequencies, possibly because of the strong relationship between age and vaccine received; participants reporting a heterologous booster dose are younger than participants reporting a homologous booster and might therefore be more likely to report reactions following vaccination (*8*).

Myocarditis is a rare adverse event associated with receipt of COVID-19 mRNA vaccines; the overall reporting rates of myocarditis following COVID-19 mRNA vaccination were highest among males aged <18 years (*5*). To date, 37 reports to VAERS of myocarditis among adults aged ≥18 years have met the case definition following administration of 81.2 million COVID-19 mRNA booster doses in the United States. One death was reported; investigation is ongoing, and other contributory factors for myocarditis are being evaluated. Among adults, the VAERS reporting rate for myocarditis following COVID-19 mRNA booster was highest (8.7 per 1 million doses administered) among men aged 18–24 years following Moderna COVID-19 booster vaccination; however, this reporting rate is lower than that following dose 2 Moderna COVID-19 vaccine for men aged 18–24 years (56.3 per 1 million doses administered) (*5*).

The findings in this report are subject to at least four limitations. First, v-safe is a voluntary program; therefore, v-safe registrants might not be representative of the entire vaccinated population (<1% of total booster recipients registered in v-safe). Second, VAERS is a passive surveillance system and subject to reporting biases and underreporting, especially of nonserious events (*4*). Third, data were insufficient to analyze COVID-19 mRNA primary series followed by a Janssen booster. Finally, assessment of myocarditis reports to VAERS received during the study period is ongoing, and counts are subject to change.

ACIP recommends that all persons aged ≥12 years receive a COVID-19 booster dose at least 5 months after receipt of dose 2 of an mRNA vaccine for the prevention of COVID-19 (*9*). Preliminary safety findings for booster vaccination from real-world settings are similar to those described in clinical trials (*6*,*7*). Vaccination providers should educate patients that local and systemic reactions are expected following a homologous COVID-19 mRNA vaccine booster. These reactions are less common than those following the second dose in the primary series. CDC and FDA will continue to monitor vaccine safety and will provide updates as needed to guide COVID-19 vaccination recommendations.

**Summary**

**What is already known about this topic?**

In preauthorization trials, adverse reactions were reported less frequently following a homologous COVID-19 mRNA vaccine booster dose than after receipt of the second primary dose.

**What is added by this report?**

Review of surveillance data found that local and systemic reactions were less frequent after a homologous COVID-19 mRNA vaccine booster dose than after the second primary vaccine dose. Myocarditis was rarely reported following an mRNA vaccine booster dose.

**What are the implications for public health practice?**

All persons aged ≥12 years should receive a COVID-19 booster dose. Vaccination providers should educate patients that local and systemic reactions are expected following a homologous COVID-19 mRNA vaccine booster; however, these reactions are less common than those following the second primary series dose.

## Footnotes

* https://covid.cdc.gov/covid-data-tracker/#vaccination-demographic

† The FDA has authorized a booster dose of either the same product administered for the primary series (homologous) or a booster dose that differs from the product administered for the primary series (heterologous). These booster authorizations apply to all three COVID-19 vaccines used in the United States: 1) Pfizer-BioNTech COVID-19 vaccine ≥5 months after dose 2 for persons aged ≥12 years, 2) Moderna COVID-19 vaccine ≥5 months after dose 2 for persons aged ≥18 years, and 3) Janssen COVID-19 vaccine ≥2 months after a single dose for persons aged ≥18 years.

§
https://www.cdc.gov/vaccines/covid-19/clinical-considerations/covid-19-vaccines-us.html

¶ Health surveys are sent for the most recent dose entered via text messages that link to web-based surveys on days 0–7 after receipt of vaccine dose; then weekly through 6 weeks after vaccination; and then at 3, 6, and 12 months after vaccination. Local injection site reactions include itching, pain, redness, and swelling. Systemic reactions include abdominal pain, myalgia, chills, diarrhea, fatigue, fever, headache, joint pain, nausea, rash, and vomiting. Health impacts include inability to perform normal daily activities, inability to work or attend school, and receipt of medical care.

** CDC and FDA encourage all health care providers to report adverse events to VAERS and are required by COVID-19 vaccine Emergency Use Authorizations to report certain adverse events after vaccination to VAERS, including death. https://vaers.hhs.gov/faq.html

†† VAERS reports are classified as serious based on the Code of Federal Regulations Title 21 (https://www.accessdata.fda.gov/scripts/cdrh/cfdocs/cfcfr/cfrsearch.cfm?fr). Reports of serious adverse events receive follow-up by VAERS staff members to obtain additional information, including medical records and, for reports of death, death certificates and autopsy reports, if available.

§§ Each VAERS report might be assigned more than one MedDRA preferred term. A MedDRA coded event does not indicate a medically confirmed diagnosis. https://www.meddra.org/how-to-use/basics/hierarchy

¶¶ Acute myocarditis was defined as presence of signs and symptoms new onset or worsening of one or more of the following signs or symptoms: chest pain, pressure, discomfort, dyspnea, shortness of breath, pain with breathing, palpitations, or syncope; or two or more of the following signs or symptoms in children aged ≤11 years: irritability, vomiting, poor feeding, tachypnea, or lethargy); and one or more new finding of elevated troponin, electrocardiogram findings consistent with myocarditis, abnormal cardiac function or wall motion on echocardiogram, cardiac magnetic resonance imaging findings consistent with myocarditis, or histopathologic findings consistent with myocarditis; and no other identifiable cause for these findings.

*** The odds of reporting an adverse event or health impact following dose 2 and booster were compared for registrants who received homologous COVID-19 mRNA vaccination using a multivariable generalized estimating equations model that accounted for the correlation between registrants and adjusted for demographic variables (i.e., age, sex, race, and ethnicity). The odds of reporting an event following homologous and heterologous booster vaccination were compared using a logistic regression model that adjusted for demographic variables of registrants; p<0.01 was considered significant. Odds ratios were not adjusted for persons who reported a primary Janssen series because of small numbers. The model did not converge for the 476 registrants who reported COVID-19 mRNA vaccination primary dose followed by a Janssen booster, and these registrants were excluded from the analysis.

††† 45 C.F.R. part 46, 21 C.F.R. part 56; 42 U.S.C. Sect. 241(d); 5 U.S.C. Sect. 552a; 44 U.S.C. Sect. 3501 et seq.

§§§
https://www.medrxiv.org/content/10.1101/2021.10.10.21264827v2
